# Magnetoelectric Current Sensors

**DOI:** 10.3390/s17061271

**Published:** 2017-06-02

**Authors:** Mirza Bichurin, Roman Petrov, Viktor Leontiev, Gennadiy Semenov, Oleg Sokolov

**Affiliations:** Department of Design and Technology of Radioequipment, Novgorod State University, Veliky Novgorod 173003, Russia; initra@yandex.ru (R.P.); viktorsergeevich.novsu@gmail.com (V.L.); Gennady.Semenov@novsu.ru (G.S.); o-v-sokolov@mail.ru (O.S.)

**Keywords:** current sensor, magnetoelectricity, magnetoelectric effect, sensor design

## Abstract

In this work a magnetoelectric (ME) current sensor design based on a magnetoelectric effect is presented and discussed. The resonant and non-resonant type of ME current sensors are considered. Theoretical calculations of the ME current sensors by the equivalent circuit method were conducted. The application of different sensors using the new effects, for example, the ME effect, is made possible with the development of new ME composites. A large number of studies conducted in the field of new composites, allowed us to obtain a high magnetostrictive-piezoelectric laminate sensitivity. An optimal ME structure composition was matched. The characterization of a non-resonant current sensor showed that in the operation range to 5 A, the sensor had a sensitivity of 0.34 V/A, non-linearity less than 1% and for a resonant current sensor in the same operation range, the sensitivity was of 0.53 V/A, non-linearity less than 0.5%.

## 1. Introduction

Current sensors are very important types of devices. There are a large number of current sensors operating on various physical principles. The most common current sensors are current transformers, magnetoresistance and Hall sensors [1,2]. Despite the fact that magnetoelectric (ME) current sensors have the small size, weight and a high sensitivity they have received relatively little attention in the publications, compared with the ME magnetic field sensors [3]. The first attempt to measure the direct current (DC) based on the ME effect was made in [4]. There are also a number of articles about ME current sensors [5,6]. Evaluation of the value of current flowing in a conductor is possible by measuring the corresponding magnetic field. The ME current sensor uses the ME effect as a basis of its measurements. As is well-known, the ME effect is a polarization response to an applied magnetic field, or conversely a magnetization response to an applied electric field. ME behavior exists as a composite effect in multiphase systems of piezoelectric and magnetostrictive materials. In a magnetostrictive-piezoelectric layered structure the interaction between magnetic and electric subsystems occurs through mechanical deformation. It means that the ME effect is much stronger at frequencies corresponding to the electromechanical resonance range. In current sensor applications the induced ME voltage coefficient is more important than the induced ME electric field coefficient, as voltage is the physical quantity measured. ME current sensors can work in different regimes. In the first case, the ME element works in a non-resonant mode regime, and in the second case, in the resonant one. The same ME element can be used as the sensitive element of the sensor in both cases. The properties of non-resonant ME current sensors were considered in [7,8], and of the resonant ones in [9]. In the paper we consider the basic principles of work of ME DC sensors of non-resonant type based on using the low-frequency ME effect and next the resonant type, working in one excited mode in the piezoelectric phase of magnetoelectric material. It should be noted that the paper describes the construction and characteristics of the ME current sensors used in practice. 

## 2. Principle of Operation

**Figure 1:**
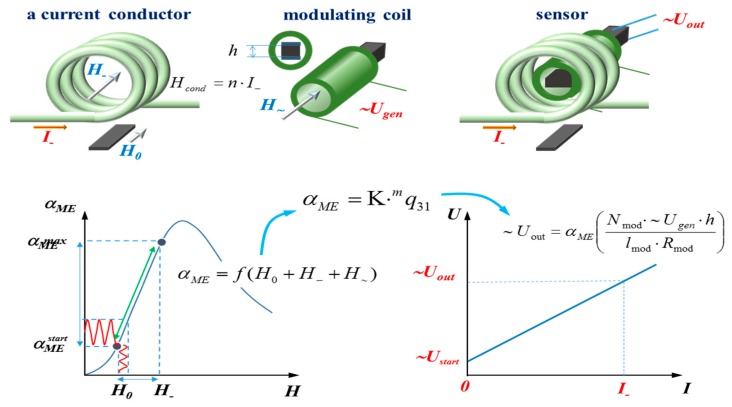
Magnetoelectric current sensor: principle of operation.

Magnetoelectric current sensor consists of a ME element made in the form of the piezoelectric plate and the side surfaces of the magnetostrictive plates are glued on. The principle of operation of ME sensor is shown in Figure 1. The figure on the left shows the dependence of dc magnetic field on the ME coefficient. The zero sensitivity of the sensor corresponds to a dc magnetic field equal to zero. The ME coefficient α_ME_ is determined by the following expression:

(1) $$\alpha_{ME}=f(H_{0}+H_{\_}+H_{\sim })$$

Passing the initial nonlinear part, we get to the linear section. In this linear section, the current sensor is working. The term α_ME_ can be written as α_ME_ = K*^m^q_31_*, where K is the gain factor, and *^m^q_31_* is the piezomagnetic module of the magnetic phase. The figure on the right shows the output characteristic of the current sensor, indicated by the red lines on the left figure. The tuning of the linear section occurs by introducing a magnetic field H_0_. Here a current conductor or a current coil replaces a bias magnetic field for inducing the ME effect. The modulating coil is needed for definition of the frequency range. Then everyone can choose to operate the linear portion of the ME voltage coefficient dependence and get a result of the sensitivity dependence for the ME sensor, that is the dependence of the output voltage on the input measuring current.

## 3. Non-Resonant Current Sensor

### 3.1. Sensor Design

**Figure 2:**
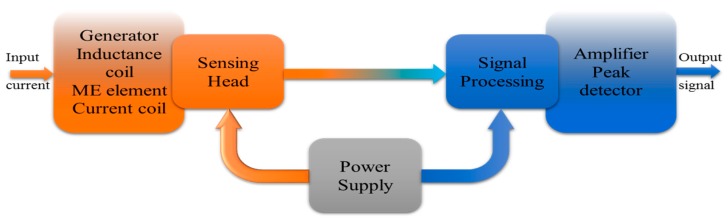
Block diagram of ME non-resonant current sensor.

The ME non-resonant current sensor consists of three major parts: the sensing head (generator, inductance coil, ME element, current coil), power supply and the signal processing (amplifier, peak detector) part. Each part affects the performance of the current sensor. The block diagram of the ME non-resonant current sensor is shown in Figure 2. The principle of operation of the sensor is based on measuring the electromotive force appearing at the output of sensitive element due to the ME effect as a result of the influence of the alternating magnetic field and a bias magnetic field.

### 3.2. Sensing Head

**Figure 3:**
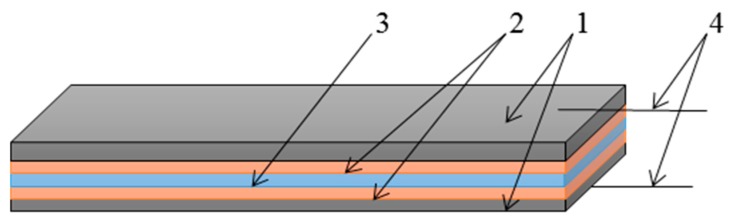
Magnetoelectric element: 1—magnetostrictive plates (Metglas); 2—adhesive layer; 3—piezoelectric plate (PZT); 4—electrodes.

The ME element, shown in Figure 3, is the primary part of the whole current sensor, and its function is to convert the measuring current into the voltage signal that can be measured easily. The ME element is the sensitive part of a ME current sensor and in our case, it consists of piezoelectric and magnetostrictive layers as shown in Figure 3. The layered structure based on a piezoceramic PZT plate in our case had thickness of 0.38 mm, length of 10 mm and width of 1 mm [8,9]. The piezoelectric was polarized in the thickness direction. The electrodes are applied on two sides of the magnetostrictive plates. Thickness of one layer of Metglas was 0.02 mm. Metglas (1) and PZT (3) plates are connected by gluing (2). Different types of glue can be used. The thickness of the adhesive layer does not exceed 10 microns. To increase the sensitivity, different numbers of Metglas layers may be used. The electrical signal is taken from the electrodes (4) and then passes on to the signal processing part of the device.

The sensitive element of the sensor is a magnetostrictive-piezoelectric composite and its magnetostriction constant is a function of magnetic field and in general can be written as λ = *f*(H_0_) [10]. In turn the piezomagnetic module depends on AC magnetic field as follows:

(2) q=dλ/qH

For the transverse ME voltage coefficient of layered composite the following dependence on material parameters was obtained [10,11]

(3) αE,31=E3H1=−V(1−V)(q11m+q21m)d31pε33p(s12m+s11m)V+ε33p(s12p+s11p)(1−V)−2pd312(1−V)

where *E_3_* and *H_1_* are the intensities of the electric and magnetic fields, *^p^s_ii_* is a coefficient of compliance of piezoelectric phase at constant electric field, *^m^s_ii_* is a coefficient of compliance of magnetic phase at constant magnetic field, *^p^ε*_33_ is a dielectric permittivity, *^p^d_31_* is a piezoelectric module, and *^m^q_ij_* is a piezomagnetic module, *V* is the volume fraction of the piezoelectric, *V = ^p^V/(^p^V + ^m^V)*, *^p^V* и *^m^V* are the volumes of the piezoelectric and magnetic phases, respectively.

The ME element is placed into the current and inductance coils, where a bias magnetic field and a variable modulation magnetic field are created, respectively.

**Figure 4:**
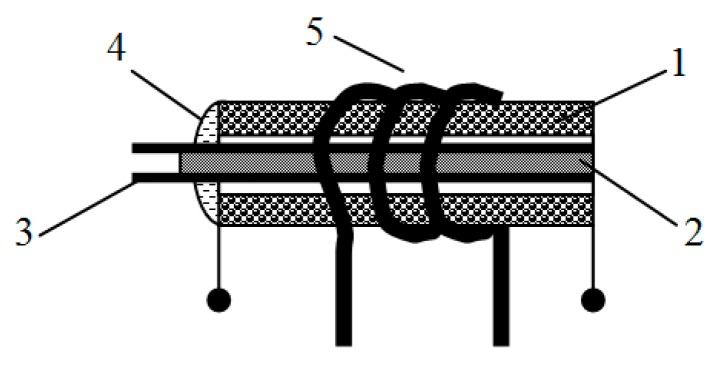
Design of the hard core of a magnetoelectric sensor: 1—inductance coil; 2—ME element; 3—leading-out wire of ME element; 4—glue; 5—current coil.

An important part is the fixation of ME element in the inductance coil. The ME element was fixed by the glue on one end of sample in order to avoid jamming the ME element surface, as shown in Figure 4.

### 3.3. Signal Processing

**Figure 5:**
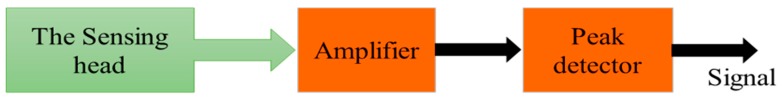
Schematic diagram of the signal processing.

The signal processing part is used to process the voltage signal from the sensing head, the block scheme is shown in Figure 5. In the non-resonant case a current sensor signal processing scheme consists of a generator that is tuned to the frequency at 500 Hz. The generator is connected to the inductance coil then the output signal from the ME element is amplified and provided to the peak detector. The current coil creates a bias magnetic field proportional to the current in the coil. For signal conversion the microprocessor also can be used. 

In the resonant case the signal processing scheme of the current sensor is similar, but the sensitivity of the scheme increases from ten to a hundred times. If the level output signal is sufficient for estimation and father processing, then it is possible to work without the amplifier.

### 3.4. Construction

**Figure 6:**
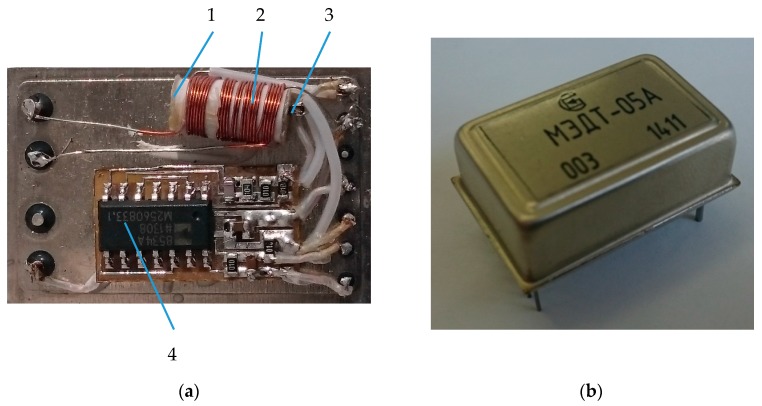
Design of a magnetoelectric non-resonant current sensor prototype: (**a**) non-resonant current sensor; (**b**) current sensor in the body.

The ME current sensor is a system consisting of the ME composite (3), a generator (4), an amplifier, a peak detector, two coils (1, 2) placed into one another and a body. Amplifier and peak detector attached to the back side of the generator. The construction of the DC ME sensor is shown in Figure 6. It can be noted that the design of an AC sensor is similar to the design of a DC sensor and differs in the presence of the conductor near the ME element. An AC sensor can also be used like a DC sensor only without the generator and modulating coil. The sensitivity of ME sensors will mainly depend on the ME properties and the maximal sensitivity should occur at the electromechanical resonance frequency.

### 3.5. Characteristics

**Figure 7:**
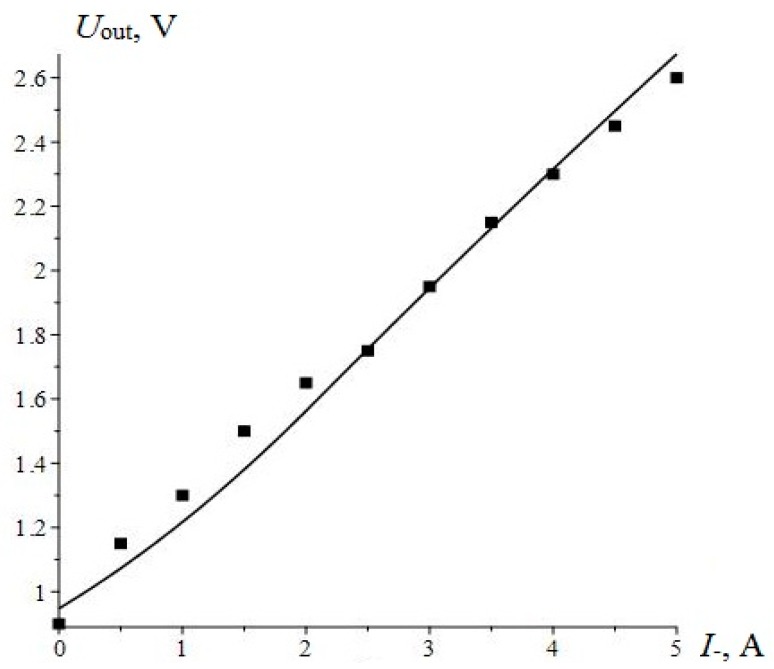
Output characteristics of the ME non-resonant current sensor.

The theoretical curve and the experimental points of the output characteristic of ME non-resonant current sensor are shown in Figure 7. The output voltage of the sensor was calculated by the following equation:

(4) Uout=KampKmνpq11L(H0+I−d)d31nUins11ms11pεε0c11Rs

where, *K_amp_* is a coefficient of amplifier, *K* is a coefficient due to manufacturing technology, *U_in_* is an input amplitude voltage, *q*_11_ is a piezomagnetic module, *c*_11_ is an effective longitudinal stiffness coefficient of the composite, *c*_11_ *= ^p^v/^p^s*_11_ *+ ^m^v/^m^s*_11_, *^p^s*_11_*, ^m^s*_11_ are the coefficients of compliance for piezoelectric and magnetostrictive phase at constant electric field, *^m^v, ^p^v* are the volume fractions of magnetostrictive and piezoelectric phases, respectively.

The term *^m^v = ^m^L/(^p^L + ^m^L) and ^p^v = ^p^L/(^p^L + ^m^L)*, where *^m^L, ^p^L* are the thicknesses of magnetostrictive and piezoelectric layers, respectively, *d_31_* is a piezoelectric module, *n* is a number of windings per unit length of the AC solenoid, *R_s_* is an active resistance of the AC solenoid, *ε* is a dielectric permittivity of piezoelectric phase, *ε_0_* is an electric constant. The calculations show suitable matching between theory and experiment. Consequently, all the parameters of the sensor are chosen quite optimally.

In our case, the magnetostrictive material Metglas based on iron alloy was used, and the dependence of ME coefficient on bias magnetic field is shown in Figure 1. The plot of the curve from α_МЕ_^start^ to α_МЕ_^max^ can be used for designing a sensor with high linearity. Measurements of Metglas parameters and calculations necessary to determine the values of bias magnetic field and the maximal measured current were made. The sensitivity of sensor reached 0.34 V/A. The working range of currents was set up to 5 A. The nonlinearity of the characteristic output was within 1%.

## 4. Resonant Current Sensor

### 4.1. Sensor Design

**Figure 8:**
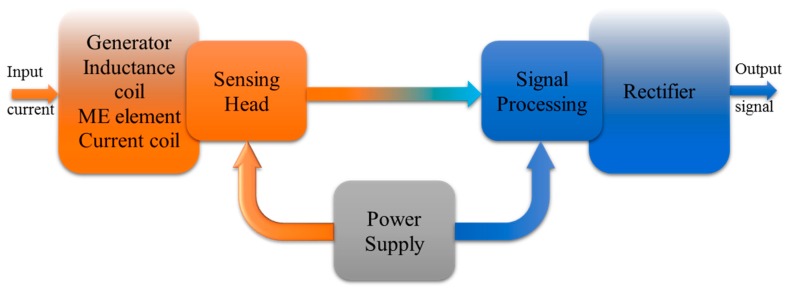
Block diagram of resonant current sensor.

The resonant current sensor has a similar design as the non-resonant one. It also consists of a generator, inductance coil, ME composite, permanent magnet, current coil (sensing head), power supply and rectifier (signal processing). The block diagram of the resonant current sensor is shown in Figure 8.

The principle of operation of the sensor is based on measuring the electromotive force appearing at the output of sensitive element due to the ME effect as a result of the influence of the alternating magnetic field and a bias magnetic field. The amplifier is not applicable here because the output signal level is enough for estimation. In this case the rectifier (diode bridge) is sufficient. 

### 4.2. Construction

**Figure 9:**
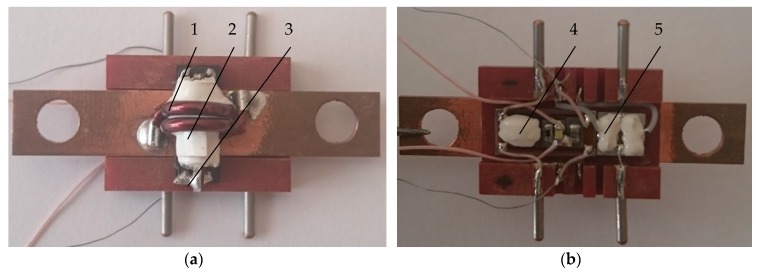
Design of magnetoelectric resonant current sensor prototype: (**a**) view from above; (**b**) bottom view.

The sensing head is similar as in the non-resonant current sensor. We used the same composite, but with a slight difference. It consists of piezoelectric and magnetostrictive layers as shown in Figure 3. The ME current sensor shown in Figure 9 consists of the ME composite (3), the generator (4), the rectifier (5), permanent magnet, the inductance (2) and current (1) coils (coils placed into one another).

The ME composite is a layered structure which includes a thin piezoceramic plate of PZT placed between two magnetostrictive layers of Metglas performing the function of electrodes. The final dimensions of the composite were 6 × 1 × 0.62 mm with a PZT concentration of 0.8. The PZT layer was poled in an electric field in the thickness direction. The ME laminate operates in a transverse mode regime in which the applied magnetic fields (H_~_, H_0_) are parallel each other and are located in the plane of the laminate. The sensing head system comprises the generator, the inductance and current coils and ME element. The generator had a frequency coincidedent with a resonance frequency of the ME composite. The coil is wound on the composite and designed to induce an AC magnetic field. The rectifier is a diode bridge and converts an AC to a DC. Such sensors can detect the AC and DC in the range from 0.01 A to 100 A depending on the design parameters.

### 4.3. Characteristics

Resonant ME current sensors were designed. The sensors work in the electromechanical range with the resonance frequency *f_res_ =* 168 kHz. The sensor sensitivity and ME voltage coefficient dependencies were investigated using the designed measurement setup. The measurement stand includes two power supplies an APS-7315 (Aktakom, Moscow, Russia) and a HMP4040 (HAMEG Instruments, Mainhausen, Germany), a HM 8112-3 multimeter (HAMEG Instruments, Mainhausen, Germany), a HMO722 oscilloscope (HAMEG Instruments, Mainhausen, Germany) and a GMW 5403 electromagnet (Magnet Systems, CA 94070, USA). The graph below shows the output characteristics of the resonant current sensor.

**Figure 10:**
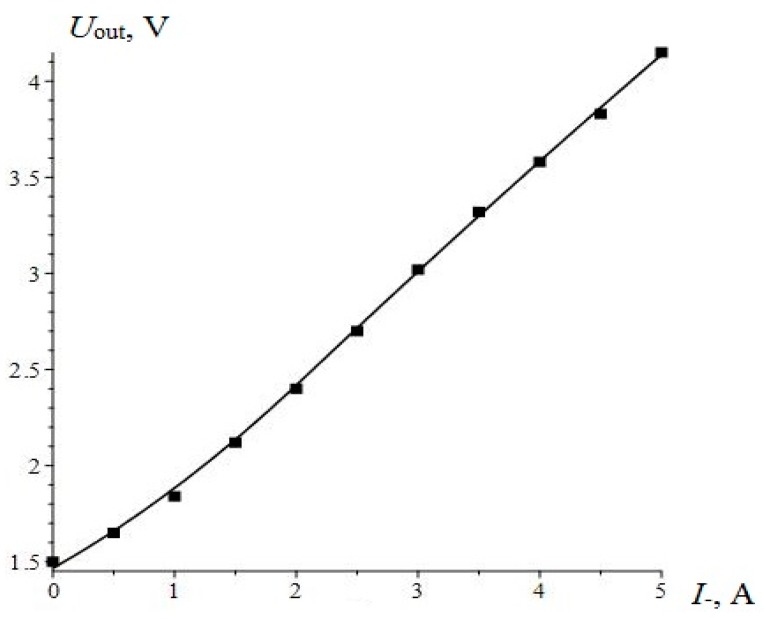
Characteristic output of the ME resonant current sensor.

Figure 10 shows the theoretical curve and the experimental points of the characteristic output of the resonant current sensor. As can be seen from the graph, the calculated and experimental results are in good agreement. This means that the selected sensor and materials parameters are optimal. The maximum linearity is reached at the additional bias field of 10 Oe. Also it is known that the sensitivity is described by the slope of the characteristic output. The sensitivity of the sensor reached 0.53 V/A. The current working range was set at 5 A. The nonlinearity of the characteristic output was within 0.5%. The resonant frequency of composite for the first axial mode was calculated by the following equation:

(5) fres=12Ls11p+rs11ms11ps11m(rpρ+ρm)

where *L* is the plate length, *^p^ρ, ^m^ρ* are the density of the piezoelectric and magnetic phases, *r* is the ratio of thicknesses, *r* = *^m^L/^p^L.*

For calculation of the characteristic output the following equation was obtained:

(6) Uout=8KQνpmq11L(H0+I−d)d31nUins11ms11pεε0c11π2(Rs+i2πfresLs)

where, *Q* is the quality factor of resonance, *f_res_* is resonance frequency, *L_s_* is the inductance of the AC solenoid.

## 5. Magnetoelectric Equivalent Circuits

### 5.1. Magnetoelectric Equivalent Circuits: Non-Resonant Current Sensor

**Figure 11:**
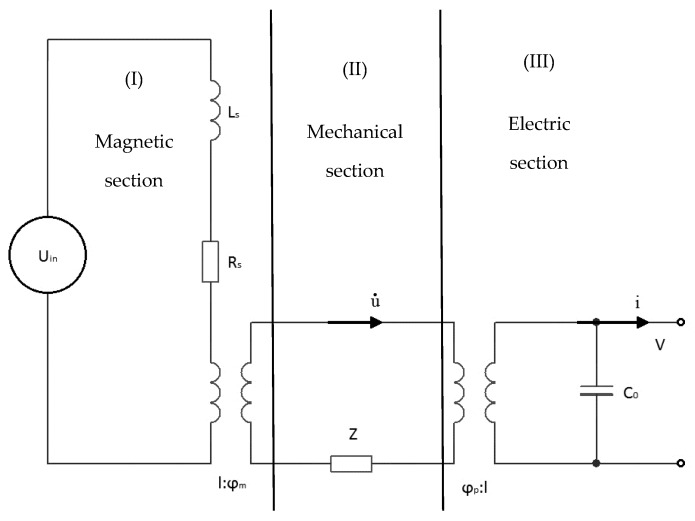
The equivalent circuit of ME non-resonant DC sensor.

For a better understanding of the current processes it is suggested to consider the magneto-electro-mechanical circuit (Figure 11), equivalent to a non-resonant current sensor [12,13]. The magnetic circuit (I) comprises an AC voltage source and an inductance coil. Flowing in the magnetic circuit (I) an electric current by means of an ideal transformer causes in mechanically circuit (II) the occurrence of mechanical current ῡ. Mechanical circuit (II) is connected with the second ideal transformer with an electric circuit (III), thus the mechanical current results in flow of electric current *i* in an electric circuit. The output voltage *V* is taken from the capacitor *C_0_* of electrical circuit. 

For the calculation we used the value of coil inductance (*L_s_ =* 5.83 × 10^−3^ H) and active resistance (*R_s_ =* 510 Ohm) obtained from the experimental data. It is necessary to make the resistance *Z* as high as possible, then the mechanical circuit will not distort the input current of the solenoid circuit. So as not to distort the mechanical connection the output circuit capacity *C_0_* must be reduced by the same amount we have increased the resistance *Z*:

(7) $$Z=-\frac{1}{2}jM\rho vA_{1}\cot\frac{kl}{2}$$

(8) C0=lb(ε33−d312s11p)Mtp

(9) M>>1

In [13], authors used the input section with the magnetic field source. In our case the input section contains an electric current. Therefore, the ideal ratio of transformation has the following form:

(10) φm=νmq11s11mA1n

For this equivalent circuit the equations are as follows:

(11) $$\begin{array}{l} R_{s}J+j\omega L_{s}J=U_{in}+\varphi_{m}\dot{u}\\ Z\dot{u}=\varphi_{m}J+\varphi_{p}V\\ J_{disp}=j\omega C_{0}V-\varphi_{p}\dot{u} \end{array}$$

Using the open-circuit conditions *J_disp_ =* 0, where *J_disp_* = (δD_3_/δt) = 0, the following equation for the output voltage was obtained:

(12) $$U_{out}=\frac{\varphi_{m}\varphi_{p}}{j\omega C_{0}\left[Z(R_{s}+j\omega L_{s})-\varphi_{m}^{2}\right]-\varphi_{p}^{2}(R_{s}+j\omega L_{s})}U_{in}$$

**Figure 12:**
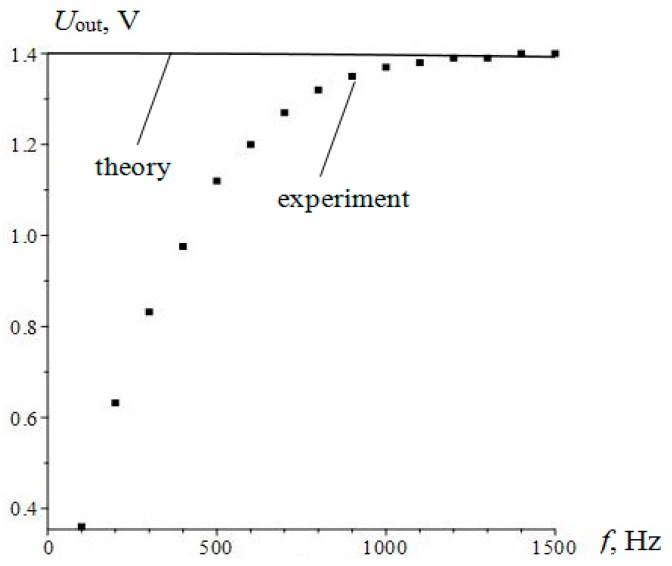
Output voltage depending on the frequency for the non-resonant current sensor.

In order to match the theoretical and experimental data, we introduced a coefficient due to manufacturing technology *K* = 0.087. In Figure 12 the theoretical curve and the experimental points of output voltage for low frequency sensor are presented.

Study of the equivalent circuit for the low-frequency mode of operation, allowed us to find the coefficient due to manufacturing technology *K*, necessary for the further consideration of the resonant mode of operation.

### 5.2. Magnetoelectric Equivalent Circuit for Resonant Current Sensor

When considering the ME equivalent circuit, we generally followed the method developed in [10]. In that paper, the authors used the magnetic circuit of the magnetic field source directly, without specifying how the magnetic field is created. In contrast, we considered the appearance of the magnetic field during the flow of electric current through the inductor. This allowed us to consider the resulting frequency response of the sensor to the complex resistance of the AC coil.

**Figure 13:**
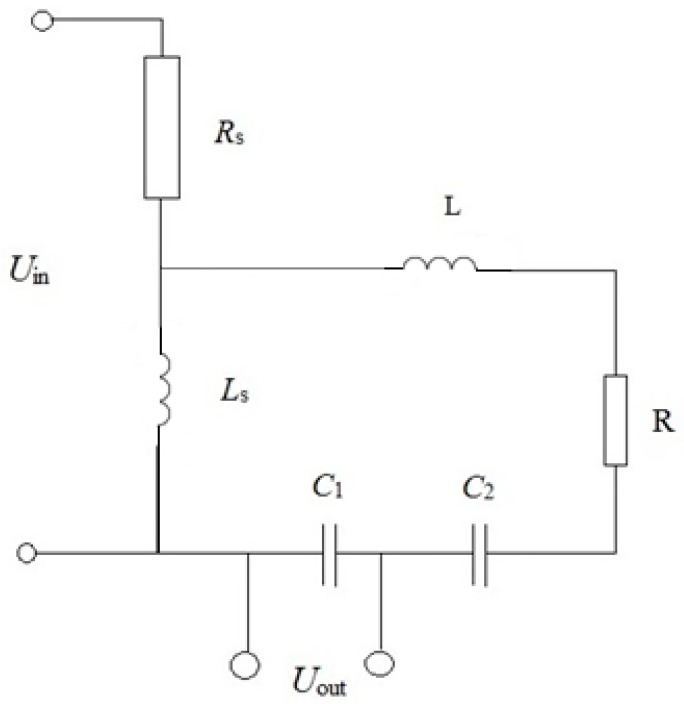
The equivalent circuit of ME resonant DC sensor.

In the study of the resonant mode of the sensor a completely simplified electrical equivalent circuit can be considered (Figure 13). The input voltage equals *U_in_*, whereby the current through the coil flows. This gives rise to a current in the resonant circuit containing the dynamic inductance of *L*, resistance *R* and capacitance *C*. The output voltage is removed from the capacitor *C_1_* constituting together with the *C_2_* capacity, full operating capacity.

ME voltage coefficient is given by the following equation:

(13) αE=E¯h1=−νmνpq11d31s11ptan(η)s11m(εε0s112pc11η+d312[tan(η)−c11s11pη])

where:

(14) $$\eta =\frac{kl}{2}$$

The magnetic field inside a long solenoid:

(15) $$h_{1}=nJ$$

where, *n* is the number of coils per unit length, *J* is a current.

Current in the solenoid:

(16) $$J=\frac{U_{in}}{R_{s}+j\omega L_{s}}$$

Output voltage:

(17) Uout=E¯(Lp+LM)=αEh1(Lp+LM)=−νPmq11Ld31s11ptan(η)s11m(εε0s112pc11η+d312[tan(η)−c11s11pη])nJ=−νpmq11Ld31s11pntan(η)Uins11m(εε0s112pc11η+d312[tan(η)−c11s11pη])(Rs+iωLs)

Taking into account the calibration coefficient K, the following ratio of voltages was found:

(18) UoutUin=−Kνpmq11Ld31s11pntan(η)s11m(εε0s112pc11η+d312[tan(η)−c11s11pη])(Rs+iωLs)

**Figure 14:**
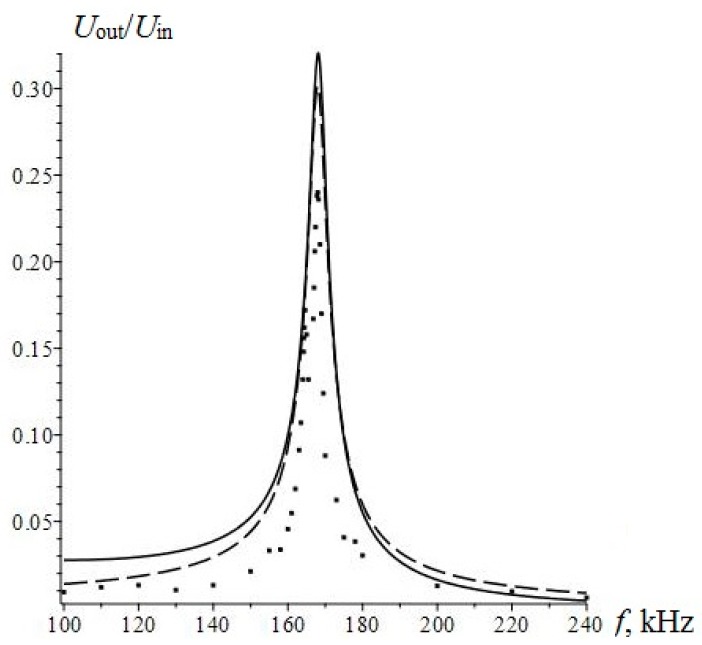
Amplitude frequency characteristics of the resonant current sensor.

The calculation and experimental data results are presented in Figure 14.

The value of the resonant frequency calculated by Equation (4) is of *f_res_ =* 168 kHz, which agrees with the experimental data. The solid line and dotted line show the results of the calculation by the equation and the equivalent circuit, respectively. The considered equivalent circuit adequately describes the current operation of the sensor in the resonant mode, as the frequency response of the equivalent circuit is in good agreement with theoretical frequency response and accurate experimental data.

## 6. Performance Comparison of Existing Current Sensors

Table 1 shows the comparative characteristics of DC current sensors. The information is about sensors produced by LEM Holding SA (HO8-NP, Freiburg, Switzerland), Honeywell Inc. (CSLW6B5, Morristown, NJ, USA), Allegro MicroSystems (ACS712ELCTR-05B-T, Worcester, MA, USA). As can be seen from the table, the ME current sensor has higher sensitivity and lower current consumption.

Known current transformers (CTs), with high breakdown voltage, are the most widely applied devices for AC sensing in traditional power systems. CTs can’t be used as DC sensors because they are based on Faraday’s law of induction. Disadvantages of CTs are: large size, high price, limited bandwidth and large consumption of metal resources, so they are only used in power stations and substations. 

Shunts are more often applied in DC converter stations and power electronics. Disadvantages of shunts are: the measured current has to be interrupted into the sensor, an overcurrent may permanently damage it, and the intrinsic inductance limits the accuracy and bandwidth. 

Hall effect current sensors are mainly applied in non-contact current measurements. The problems of the Hall effect sensors are: low sensitivity, low breakdown voltage, and susceptibility to temperature, which limits them to applications in high voltage power systems [2]. 

Compared with the current sensors presented above, the ME current sensor has advantages of higher sensitivity, higher linearity, lower cost, simple structure of sensor, which make it most promising for current measurements.

## 7. Conclusions

In this paper the principle of operation of ME current sensors was considered. Theoretical and experimental investigations of both low frequency and resonant current sensors were discussed. The optimal materials for sensitive elements were chosen and PZT and Metglas were used, respectively in the the piezoelectric and magnetostrictive phases. The equivalent circuit method was used to calculate the characteristics of the sensors. The calculation results allowed us to estimate the internal parameters. The design and development of non-resonant and resonant current sensors were discussed. The characterization of the non-resonant current sensor showed that in the operation range up to 5 A, its sensitivity was 0.34 V/A, and its non-linearity was less than 1% and for the resonant one in the same operation range, the sensitivity was 0.53 V/A, and the non-linearity less than 0.5%. The resonant current sensor has higher sensitivity compared with the non-resonant current sensor, therefore it is recommended for the detection of very low currents, for example, leakage currents. Finally, ME current sensors can be used for measuring equipment, power nets and control systems, security systems and safety systems; in metal detectors and the automotive industry, rail transport; in wireless electricity metering systems and space technology and robotics. 

## Figures and Tables

**Table 1:** Performance comparison of current sensors.

Sensors	HO8-NP	CSLW6B5	ACS712ELCTR-05B-T	Magnetoelectric Sensor
Measuring principle	Hall effect measuring principle	Miniature ratiometric linear Hall effect sensor	Hall effect sensor	Magnetoelectric effect
Primary current, measuring range, I_pm_ (A)	0–20	±5	±5	0–5
Sensitivity (V/A)	0.1	0.2	0.185	0.53
Supply voltage (V)	5 ± 10%	4.5–10.5	5 ± 10%	5 ± 10%
Current consumptions (mA)	19	9	10	2.5
Accuracy (%)	1	0.5	1.5	0.5
Output voltage range U_out_ (V)	2.5−0.5	2.7–3.7	2.5–4.5	1.5–4.5
Size (mm)	24 × 12 × 12	16.2 × 14 × 10	6 × 5 × 1.75	30 × 20 × 10

## References

[1] Pavel R., Alois T. (2007). Modern Sensors Handbook.

[2] Ouyang Y., He J., Hu J., Wang S.X. (2012). A current sensor based on the giant magnetoresistance effect: Design and potential smart grid applications. Sensors.

[3] Bichurin M.I., Petrov V.M., Petrov R.V., Tatarenko A.S., Grosz A., Michael J.H.-S., Subhas C.M. (2016). Magnetoelectric magnetometers. High Sensitivity Magnetometers.

[4] Bichurin M.I., Petrov V.M., Petrov R.V., Kiliba Y.V., Bukashev F.I., Smirnov A.Y., Eliseev D.N. (2002). Magnetoelectric sensor of magnetic field. Ferroelectrics.

[5] Palneedi H., Annapureddy V., Priya S., Ryu J. (2016). Status and Perspectives of Multiferroic Magnetoelectric Composite Materials and Applications. Actuators.

[6] Lu C., Li P., Wen Y., Yang A., Yang C., Wang D., He W., Zhang J. (2014). Magnetoelectric Composite Metglas/PZT-Based Current Sensor. IEEE Trans. Magn..

[7] Petrov R.V., Yegerev N.V., Bichurin M.I., Aleksić S.R. Current sensor based on magnetoelectric effect. Proceedings of the 18th International Symposium on Electrical Apparatus and Technologies (SIELA).

[8] Solovyev I.N., Solovyev A.N., Petrov R.V., Bichurin M.I., Vučković A.N., Raičević N.B. Sensitivity of Magnetoelectric Current Sensor. Proceedings of the 11th International Conference on Applied Electromagnetics—ΠEC 2013.

[9] Petrov R.V., Solovyev I.N., Soloviev A.N., Bichurin M.I. Magnetoelectic current sensor. Proceedings of the PIERS Proceedings.

[10] Bichurin M.I., Petrov V.M. (2014). Modeling of Magnetoelectric Effects in Composites. Springer Ser. Mater. Sci..

[11] Bichurin M., Viehland D. (2011). Magnetoelectricity in Composites.

[12] Balabanian N. (2012). Fundamentals of Circuit Theory.

[13] Dong S.X., Zhai J.Y. (2008). Equivalent circuit method for static and dynamic analysis of magnetoelectric laminated composites. Chin. Sci. Bull..

